# ﻿Molecular cytogenetic characterization of 9 populations of four species in the genus *Polygonatum* (Asparagaceae)

**DOI:** 10.3897/compcytogen.18.122399

**Published:** 2024-05-16

**Authors:** Yan-Fang Wei, Xiang-Hui Jiang, Rong Song, Chao-Wen She

**Affiliations:** 1 College of Life Sciences and Chemistry, Hunan University of Technology, Zhuzhou, Hunan, 412007, China Hunan University of Technology Zhuzhou China; 2 Key Laboratory of Research and Utilization of Ethnomedicinal Plant Resources of Hunan Province, Huaihua University, Huaihua, Hunan, 418008, China Huaihua University Huaihua China; 3 Institute of Agricultural Environment and Ecology, Hunan Academy of Agricultural Sciences, Changsha, Hunan, 410125, China Institute of Agricultural Environment and Ecology, Hunan Academy of Agricultural Sciences Changsha China

**Keywords:** Cytotaxonomy, fluorescence *in situ* hybridization, karyotype, karyotype asymmetry, *Polygonatum*, ribosomal RNA genes (rDNA)

## Abstract

To characterize the chromosomes of the four species of *Polygonatum* Miller, 1754, used in traditional Chinese medicine, *P.cyrtonema* Hua, 1892, *P.kingianum* Collett et Hemsley, 1890, *P.odoratum* (Miller, 1768) Druce, 1906, and *P.sibiricum* Redouté, 1811, and have an insight into the karyotype variation of the genus *Polygonatum*, fluorescence *in situ* hybridization (FISH) with 5S and 45S rDNA oligonucleotide probes was applied to analyze the karyotypes of 9 populations of the four species. Detailed molecular cytogenetic karyotypes of the 9 populations were established for the first time using the dataset of chromosome measurements and FISH signals of 5S and 45S rDNA. Four karyotype asymmetry indices, CV_CI_, CV_CL_, M_CA_ and Stebbins’ category, were measured to elucidate the asymmetry of the karyotypes and karyological relationships among species. Comparison of their karyotypes revealed distinct variations in the karyotypic parameters and rDNA patterns among and within species. The basic chromosome numbers detected were *x* = 9, 11 and 13 for *P.cyrtonema*, *x* = 15 for *P.kingianum*, *x* = 10 and 11 for *P.odoratum*, and *x* = 12 for *P.sibiricum*. The original basic chromosome numbers of the four species were inferred on the basis of the data of this study and previous reports. All the 9 karyotypes were of moderate asymmetry and composed of metacentric, submetacentric and subtelocentric chromosomes or consisted of two of these types of chromosomes. Seven populations have one locus of 5S rDNA and two loci of 45S rDNA, and two populations added one 5S or 45S locus. The karyological relationships among the four species revealed by comparison of rDNA patterns and PCoA based on *x*, 2*n*, TCL, CV_CI_, M_CA_ and CV_CL_ were basically accordant with the phylogenetic relationships revealed by molecular phylogenetic studies. The mechanisms of both intra- and inter-specific dysploidy in *Polygonatum* were discussed based on the data of this study and literature.

## ﻿Introduction

The genus *Polygonatum* Miller, 1754, as the largest genus in the tribe Polygonateae (Asparagaceae), comprises ca. 70 species (Chen and Tamura 2000). The genus is distributed throughout the temperate regions of the Northern Hemisphere with ca. 50 species in east Asia (from Himalaya to China and Japan), 5 species in Europe and 3 species in North America, and main diversification centered in southwest China and northeast Asia (Chen and Tamura 2000; Meng et al. 2014; Wang et al. 2022). *Polygonatum* is also one of the most important medicinal taxa in Asia. At least 37 species and 1 variety of *Polygonatum* plants have been used as traditional medicine and functional food with the rhizome being the most commonly used part of the plant (Zhao et al. 2018). In traditional Chinese medicine (TCM), the dry rhizome of *P.odoratum* (Miller, 1768) Druce, 1906 is known as Yuzhu (Polygonati Odorati Rhizoma), while the dry rhizomes of *P.sibiricum* Redouté, 1811, *P.kingianum* Collett et Hemsley, 1890, and *P.cyrtonema* Hua, 1892, are known as Huangjing (Polygonati Rhizoma) (Chinese Pharmacopoeia Commission 2020). They are both Yin-nourishing herbs that are associated with delaying senescence and are often used to treat osteoporosis, feebleness, fatigue, diabetes and lung disorders (Zhao et al. 2018; Chinese Pharmacopoeia Commission 2020).

*Polygonatum* species show a high variation in morphology and a wide overlap in geographical distribution, which makes infrageneric classification and species identification very complicated (Tang 1978; Chen and Tamura 2000; Meng et al. 2014). Since the middle of the last century, much conventional cytogenetic work has been conducted to reveal cytotaxonomic relationships and evolutionary trends of karyotype within the genus (Suomalainen 1947; Therman 1953; Kumar 1959; Mehra and Pathania 1960; Kawano and Iltis 1963; Inoue 1965; Mehra and Sachdeva 1976; Kim and Kim 1979; Fang et al. 1984; Wang et al. 1987, 1991; Yang et al. 1988, 1992; Chen et al. 1989; Hong and Zhu 1990; Tamura 1990, 1993; Wang et al. 1993; Shao et al. 1993, 1994; Han et al. 1998; Wu et al. 2001; Weiss-Schneeweiss and Jang 2003; Chen and Zhou 2005; Deng et al. 2009; Zhao et al. 2014; Zhou et al. 2020). Conventional karyotyping revealed significant variation in basic chromosome number among species in the genus, dysploid variation within species, and bimodality of karyotypes of most populations of *Polygonatum* species studied (Wang et al. 1987, 1991; Yang et al. 1992; Chen and Zhou 2005; Deng et al. 2009; Zhao et al. 2014; Wang et al. 2016a; Zhou et al. 2020).

The classification of *Polygonatum* has long been controversial. Baker (1875) classified *Polygonatum* into three sections based on its leaf arrangement, section Alternifolia, section Verticillata, and section Oppositifolia. Tang (1978) divided *Polygonatum* into eight series based on more detailed morphological characters. Tamura (1993) proposed a new classification on the basis of a combination of cytogenetics and morphology, dividing the genus into two sections, section Polygonatum (basic chromosome number: *x* = 9, 10, 11) and section Verticillata (*x* = 14 or 15). The most recent and widely accepted classification is that of Meng et al. (2014) who divided the genus into three sections based on molecular phylogenetic and morphological evidence: (i) sect. Polygonatum including species with alternate leaves and *x* = 9–11, (ii) sect. Sibirica including species with whorled leaves and *x* = 12, and (iii) sect. Verticillata including species with variable phyllotaxy and *x* = 13–15. This infrageneric classification system was confirmed by several subsequent molecular phylogenetic studies (Floden and Schilling 2018; Zhao et al. 2019; Xia et al. 2022; Wang et al. 2022; Qin et al. 2024). However, this classification has not been validated by molecular cytogenetics. To date, more than 50 species of *Polygonatum* have been conventionally karyotyped (Zhao et al. 2014). These karyotype analyses can only provide limited information on species identification and karyotype evolution among *Polygonatum* species due to a lack of effective markers. Although FISH (fluorescence *in situ* hybridization) technology has been widely used in genome analysis of plants (Jiang and Gill 2006), there have not been any report of chromosome characterization of *Polygonatum* species using FISH.

The ribosomal genes, 45S (18S-5.8S-26S) and 5S rDNAs, are organized in tandem arrays with high copy numbers, and then widely utilized as probes for FISH in plants. The rDNA FISH signals can be used as informative markers for a better characterization of the chromosomes of plant species, revealing genome organization at molecular cytogenetic level (e.g. Moscone et al. 1999; Chacón et al. 2012; She et al. 2015; Mitrenina et al. 2023). Furthermore, comparison of rDNA patterns (namely the number and location of 5S and 45S rDNA loci) among species within a genus contributes to the understanding of the mechanism of chromosome evolution and phylogenetic relationships between related species (e.g. Moscone et al. 2007; Chacón et al. 2012; Siljak-Yakovlev and Peruzzi 2012; She et al. 2015, 2017, 2020; Senderowicz et al. 2022; Yucel et al. 2022; Mitrenina et al. 2023). However, to date, there has been no molecular cytogenetic kayotype analysis of *Polygonatum* species except for the report of FISH detection of 45S rDNA in *P.odoratum* and *P.cyrtonema* (Wu et al. 2001).

In the present study, comparative molecular cytogenetic analysis of 9 populations of four *Polygonatum* species, *P.cyrtonema*, *P.kingianum*, *P.sibiricum* and *P.odoratum*, was conducted using dual-color FISH with 5S and 45S rDNA oligonucleotide probes. Detailed molecular cytogenetic karyotypes of these populations were quantitatively established using a combination of chromosome measurements and rDNA FISH signals. Four different karyotype asymmetry indices of each population were calculated for evaluating asymmetry of the karyotypes and karyological relationships among the populations. The combined data of karyotypic parameters and rDNA patterns were assessed to gain insights into the intra- and inter-specific karyotype differentiation as well as the phylogenetic relationships among the four species.

## ﻿Material and methods

### ﻿Plant material

Plants of 9 populations including four of *P.cyrtonema*, two of *P.kingianum*, two of *P.odoratum* and one of *P.sibiricum* (Suppl. material 1: table S1) were collected from different regions of China, and cultivated in Huangjing germplasm gardens of Agricultural Environment and Ecology Institute of Hunan Academy of Agricultural Sciences. The plants were identified by Dr. Rong Song of Agricultural Environment and Ecology Institute of Hunan Academy of Agricultural Sciences.

### ﻿Chromosome preparation

The rhizomes used for cytogenetic experiments were cultivated in pots with mixed planting soil consisting of humus soil and sandy soil, and young new roots grew from the rhizomes in about 10 to 14 days. Chromosome spreads were prepared using a protocol previously published by us (She et al. 2015) with minor modifications. Root tips were harvested and treated with saturated α-bromonaphthalene at 28 °C for 5.0 h, and then fixed in 3:1 (v/v) methanol/glacial acetic acid overnight at 4 °C. The fixed root tips were thoroughly rinsed in deionized water and digested in a mixture of 1% cellulase RS and 1% pectolyase Y23 (Yakult Pharmaceutical Industry Co., Ltd. Tokyo, Japan) in citric buffer (pH 4.5) at 37 °C for 2 h. The enzyme solution was replaced by deionized water. The digested root tips were transferred to a glass slide and mashed by using fine-pointed forceps with the fixative solution. Then, the slides were flame-dried. The slides with well-spread metaphase chromosomes were selected under a Olympus BX51 phase contrast microscope and stored at -20 °C until use.

### ﻿Probe DNA preparation

The 5S rDNA oligonucleotide probes 5S-1 and 5S-2 and the 45S rDNA oligonucleotide probes 45S-1, 45S-2 and 45S-3, which were described previously by Han et al. (2018), were synthesized by Sangon Bioengineering Co., LTD (Shanghai, China). 5S-1 and 5S-2 were labeled with 6-carboxyl fluorescein (6-FAM) at the 5’-terminus and then mixed together to make the 5S rDNA probe solution. 45S-1, 45S-2, and 45S-3 were labeled with 6-carboxyl-tetramethyl rhodamine (TAMRA) at the 5’-terminus and mixed together to make the 45S rDNA probe solution.

### ﻿FISH and signal detection

FISH was performed according to the procedure described by Han et al. (2018). The hybridization solution (each slide) was as follows: deionized formamide, 10 µL; 50% dextran sulphate, 4 µL; 20 × SSC, 2 µL; salmon sperm DNA, 2 µL (40 ng); 5S rDNA probe, 1 µL (40 ng); 45S rDNA probe, 1 µL (40 ng). The slides were baked at 65 °C for 45 min, cooled, and then denatured in 70% deionized formamide at 85 °C for 2.5 min. Further, they were dehydrated in 70%, 90%, and 100% alcohol series each for 5 min at −20 °C, followed by air drying. The hybridization solution was poured onto the denatured chromosome slide and then incubated in a moist box infiltrated by 2 × SSC at 37 °C overnight.

The slides were washed in 2 × SSC twice each for 5 min at room temperature after hybridization. Then, the chromosomes were counterstained with 3 µg ml^−1^ DAPI in 30% (v/v) Vectashield H-1000 and visualized with an Olympus BX60 microscope equipped with a QImaging Retiga R6 CCD camera (Teledyne Photometrics, Canada) which was controlled using Ocular software (Teledyne Photometrics, Canada). Observations were made using UV, blue and green excitation filters for DAPI, 6-FAM, and TAMRA, respectively. Grey-scale images were digitally captured and merged by the Ocular software. The final images were adjusted with Adobe Photoshop CS 8.01.

### ﻿Karyotype analysis

The methodology of karyotype analysis described recently by us was used (She et al. 2023). For each population, five metaphase cells with high condensation were selected for measurement using Adobe Photoshop CS 8.01. The length of long arm (L) and short arm (S) of each chromosome and the length between the center of FISH signal and centromere were measured. For numerically characterizing the karyotypes, the following parameters were calculated: (i) chromosome relative lengths (RL, % of haploid complement); (ii) arm ratios (AR = L/S); (iii) total chromosome length of the haploid complement (TCL; i.e. the karyotype length); (iv) mean chromosome length (C); (v) percent distance from the centromere to the rDNA locus; (vi) mean centromeric index (CI); (vii) Four karyotype asymmetry indices including coefficient of variation (CV) of centromeric index (CV_CI_), coefficient of variation (CV) of chromosome lengths (CV_CL_), mean centromeric asymmetry (M_CA_) and Stebbins’ asymmetry category. The meaning and calculation formulae of these indices refer to Paszko (2006) and Peruzzi and Eroglu (2013). The chromosomes were classified as metacentric (m), submetacentric (sm), subtelocentric (st) and telocentric (t) according to arm ratio (Levan et al. 1964). The chromosomes were arranged in order of decreasing length. Idiograms were drawn based on the dataset of chromosome measurements as well as the location and size of rDNA-FISH signals.

Bidimensional scatter diagram for the 9 populations with M_CA_ vs. CV_CL_ was plotted in order to visualize karyotype asymmetry relationships among them. To determine the karyological relationships among the 9 populations, a principal coordinate analysis (PCoA) using Gower’s similarity coefficient were performed based on six quantitative parameters (*x*, 2*n*, TCL, CV_CI_, M_CA_ and CV_CL_) according to the proposal by Peruzzi and Altınordu (2014).

## ﻿Results

### ﻿General karyotype features

**Figure 1.**
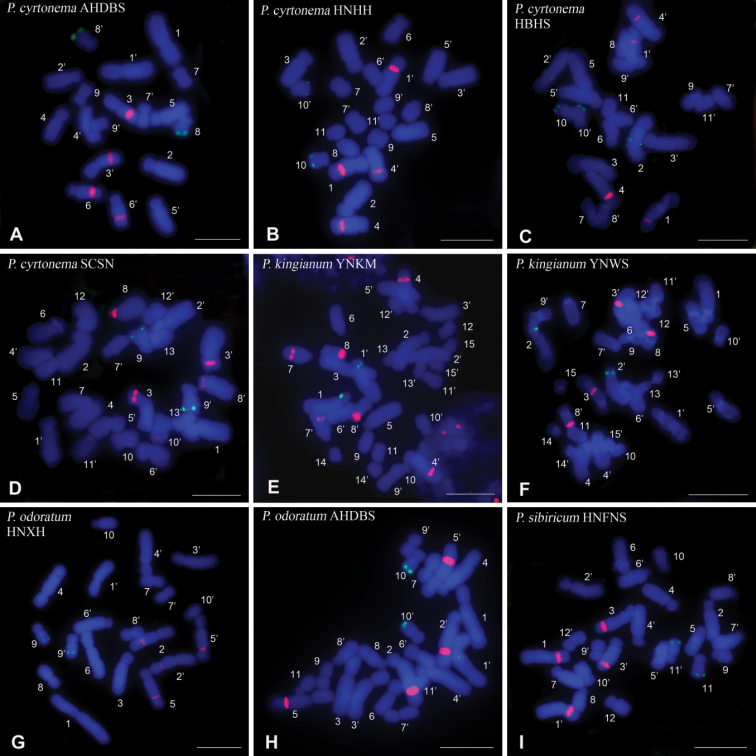
FISH to metaphase chromosomes of 9 populations of four *Polygonatum* species, *P.cyrtonema* (Pc), *P.kingianum* (Pk), *P.odoratum* (Po) and *P.sibiricum* (Ps), using 5S rDNA (green) and 45S rDNA (red) oligonucleotide probes. Chromosomes were counterstained with DAPI (blue). The chromosome numbers were designated by karyotyping **A**Pc AHDBS **B**Pc HNHH **C**Pc HBHS **D**Pc SCSN **E**Pk YNKM **F**Pk YNWS **G**Po HNXH **H**Po AHDBS **I**Ps HNFNS. Scale bars: 10 µm.

**Figure 2.**
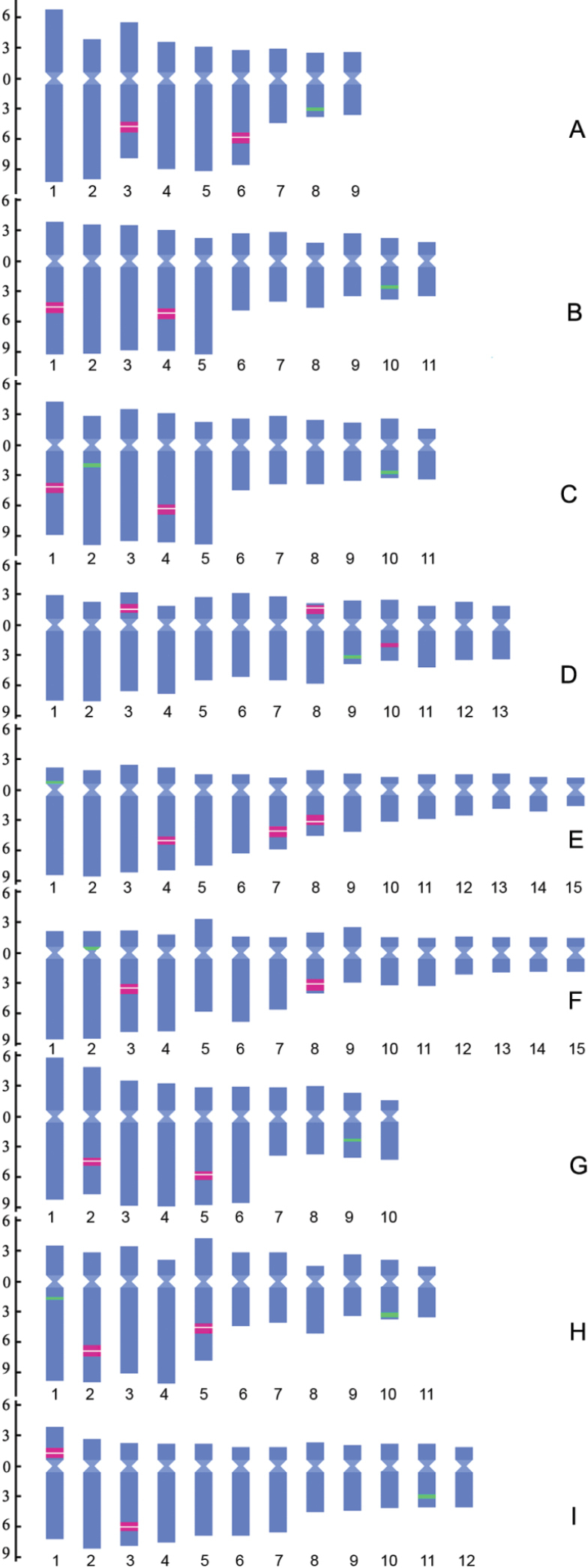
Idiograms of 9 populations of four *Polygonatum* species, *P.cyrtonema* (Pc), *P.kingianum* (Pk), *P.odoratum* (Po) and *P.sibiricum* (Ps), that display the chromosome measurements, and the location and size of the 5S (green) and 45S (red) rDNA FISH signals **A**Pc AHDBS **B**Pc HNHH **C**Pc HBHS **D**Pc SCSN **E**Pk YNKM **F**Pk YNWS **G**Po HNXH **H**Po AHDBS **I**Ps HNFNS. The ordinate scale on the left indicates the relative length of the chromosomes (i.e. % of haploid complement). The numbers at the bottom indicate the the serial number of chromosomes.

The general karyotype features of the 9 populations of *P.cyrtonema*, *P.kingianum*, *P.odoratum* and *P.sibiricum* are listed in Table 1. The measurement data of the chromosomes of each population are given in Suppl. material 2: table S2. Representative mitotic chromosomes hybridized with the 5S and 45S rDNA probes are shown in Fig. 1. The idiograms displaying the chromosome measurements as well as the location and size of rDNA FISH signals are illustrated in Fig. 2.

**Table 1.** Karyotype parameters of 9 populations of four *Polygonatum* species.

Populations*	Karyotype formula (KF)	TCL ± SE (μm)	C (μm)	RRL	CI ± SE	CV_CI_	CV_CL_	M_CA_	Stebinns’ types
Pc AHDBS	2*n* = 18 = 10m(2SAT) + 6sm + 2st(2SAT)	72.23 ± 8.83	8.03	6.23–17.01	33.45 ± 7.10	21.99	34.38	31.46	2B
Pc HNHH	2*n* = 22 = 4m + 16sm(4SAT) + 2st	80.34 ± 20.91	7.30	5.46–11.95	31.84 ± 7.33	23.02	34.85	36.28	3B
Pc HBHS	2*n* = 22 = 6m + 10sm(2SAT) + 6st(2SAT)	72.39 ± 5.03	6.58	5.01–13.08	32.46 ± 8.32	24.59	38.51	35.07	3B
Pc SCSN	2*n* = 26 = 8m + 14sm(4SAT) + 4st	84.07 ± 6.93	6.47	5.21–10.41	32.22 ± 6.25	19.40	22.94	35.56	3A
Pk YNKM	2*n* = 30 = 6m + 10sm(2SAT) + 14st(4SAT)	93.40 ± 12.14	6.23	2.85–10.13	28.05 ± 9.51	33.92	43.90	43.90	3B
Pk YNWS	2*n* = 30 = 10m + 8sm(2SAT) + 12st(2SAT)	80.38 ± 6.61	5.36	3.34–10.61	31.26 ± 10.62	33.98	42.16	37.48	3B
Po HNXH	2*n* = 20 = 8m(2SAT) + 12sm(2SAT)	86.32 ± 8.18	8.63	5.95–11.61	33.86 ± 7.79	23.00	31.47	32.28	2A
Po AHDBS	2*n* = 22 = 6m + 10sm(2SAT) + 6st(2SAT)	88.61 ± 10.32	8.01	4.90–12.82	30.90 ± 8.85	28.65	36.88	38.20	3B
Ps HNFNS	2*n* = 24 = 12sm(2SAT) + 12st(2SAT)	76.14 ± 5.30	6.35	5.91–11.16	28.30 ± 5.85	20.67	23.61	43.40	3A

* Pc = *P.cyrtonema*, Pk = *P.kingianum*, Po = *P.odoratum*, Ps = *P.sibiricum*.

The four populations of *P.cyrtonema* have three different chromosome numbers: 2*n* = 18 for Pc AHDBS, 2*n* = 22 for Pc HNHH and Pc HBHS, and 2*n* = 26 for Pc SCSN, among which 2*n* = 26 is reported for the first time. Both populations of *P.kingianum* have the same chromosome number 2*n* = 30. The chromosome numbers of the two populations of *P.odoratum* are different: 2*n* = 20 for Po HNXH, 2*n* = 22 for Po AHDBS. The chromosome number of *P.sibiricum* is 2*n* = 24. Among the 9 populations, the total length of the haploid complement (TCL) ranges from 72.23 μm (Pc AHDBS) μm to 93.40 μm (Pk YNKM) with a mean chromosome length between 5.36 μm (Pk YNWS) and 8.63 μm (Po HNXH), showing both inter- and intra-specific variation. According to the classification of Lima-de-Faria (1980), the metaphase chromosomes of the four *Polygonatum* species are of medium size. In regard to range of relative length (RRL), the smallest RRL is observed in Ps HNFNS (5.91–11.16), while the largest RRL is showed in Pk YNKM (2.85–10.13). That is, Ps HNFNS and Pk YNKM exhibit the smallest and the largest variation in chromosome length, respectively. The mean centromeric index (CI) of the chromosome complements varies between 33.86 ± 7.79 (Po HNXH) and 28.05 ± 9.51 (Pk YNKM). That is, Po HNXH and Pk YNKM are characterized by the smallest and the largest level of variation in the centromeric index, respectively.

The karyotypes are composed of m, sm and st chromosomes or consisted of two of these types of chromosomes (Table 1, Suppl. material 2: table S2; Fig. 2). The karyotype formulas are different among populations. This is true even in the populations of the same species with the same number of chromosomes. In Po HNXH, the lengths of the homologous chromosomes of pairs 1 and 6 differ significantly, exhibiting heterozygosity in chromosomal morphology (Fig. 1G). There are clear gaps in chromosome length between the 6^th^ and 7^th^ pair in Pc AHDBS and Po HNXH, between the 5^th^ and 6^th^ pair in Pc HNHH, Pc HBHS and Po AHDBS, exhibiting distinct bimodal karyotypes (Fig. 2A, B, C, G, H; Suppl. material 2: table S2). The difference between the relative lengths of the chromosomes on either side of the gaps is 3.9–5.17 (Suppl. material 2: table S2). The bimodal karyotype can be described as consisting of several pairs of large chromosomes and several pairs of small chromosomes (large + small). If so, the constitutions of the bimodal karyotypes of Pc AHDBS, Pc HNHH, Pc HBHS, Po HNXH and Po AHDBS are 6 + 3, 5 + 6, 5 + 6, 6 + 4, 5 + 6, respectively. Unusually, Pc SCSN has only a small gap in chromosome length between the 8^th^ and 9^th^ pair, showing indistinct bimodality (Fig. 2D; Suppl. material 2: table S2). In Pk YNKM and Pk YNWS, only small gaps in chromosome length between 9^th^ and 10^th^ pair exist, and four pairs of chromosomes are very short and of similar length (pairs 12 to 15), showing indistinct bimodality (Fig. 2E, F; Suppl. material 2: table S2). Ps HNFNS has a small gap between the 7^th^ and 8^th^ pair, also showing indistinct bimodality (Fig. 2I; Suppl. material 2: table S2). Different numbers and locations of secondary constrictions (SCs) are observed in the 9 populations (Fig. 2, Suppl. material 3: fig. S1). All the four populations of *P.cyrtonema* show four SCs, which are located on the long arms of the 3^rd^ and 6^th^ pairs in Pc AHDBS, on the long arms of the 1^st^ and 4^th^ pairs in Pc HNHH and Pc HBHS, and on the short arms of the 3^rd^ and 8^th^ pairs in Pc SCSN (Fig. 2A, B, C, D, Suppl. material 3: fig. S1A, B, C, D). Pk YNKM had six SCs which are located on the long arms of the 4^th^ , 7^th^ and 8^th^ pairs, while Pk YNWS had four SCs which are located on the long arms of the 3^rd^ and 8^th^ pairs (Fig. 2E, F, Suppl. material 3: fig. S1E, F). Both Po HNXH and Po AHDBS have four SCs which are situated on the long arms of the 2^nd^ and 5^th^ pairs (Fig. 2G, H, Suppl. material 3: fig. S1G, H). In Ps HNFNS, two SCs is found on the short arms of the 1^st^ pair, and another two SCs are located on the long arms of the 3^rd^ pair (Fig. 2I, Suppl. material 3: fig. S1I).

The values of the four karyotype asymmetry indices including CV_CI_, CV_CL_, M_CA_ and Stebbins’ type are presented in Table 1. According to the critical review by Peruzzi and Eroglu 2013, CV_CI_ is the measure of the heterogeneity of centromere position, CV_CL_ is a powerful statistical parameter for estimating the interchromosomal asymmetry, and M_CA_ is the most appropriate parameter for characterizing the intrachromosomal asymmetry. The ranges of CV_CI_, CV_CL_ and M_CA_ are as follows: CV_CI_ = 19.40 (Pc SCSN) – 33.98 (Pk YNWS), CV_CL_ = 22.94 (Pc SCSN) – 43.90 (Pk YNKM), M_CA_ = 31.46 (Pc AHDBS) – 43.90 (Pk YNKM). The CV_CL_ values reveal that Pc SCSN and Pk YNKM have the least and the most asymmetric karyotype, respectively, among the 9 populations in terms of interchromosomal asymmetry. The M_CA_ values reveal that Pc AHDBS and Pk YNKM have the lowest and the highest intrachromosomal asymmetry, respectively. With respect to the Stebbins’ type, these karyotypes fall into 2A, 2B, 3A or 3B categories, possessing moderate degree of asymmetry (Stebbins 1971).

**Figure 3.**
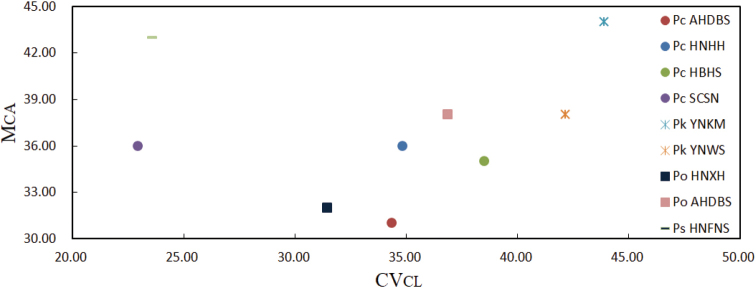
Bidimensional scatter plot of M_CA_ vs. CV_CL_ for the 9 populations of four *Polygonatum* species, *P.cyrtonema* (Pc), *P.kingianum* (Pk), *P.odoratum* (Po) and *P.sibiricum* (Ps).

The karyotype asymmetry relationships among the 9 populations that are expressed by means of bidimensional scatter plot of M_CA_ vs. CV_CL_ are illustrated in Fig. 3. It is obvious that the karyotpye structure of these populations can be discriminated by these two parameters. As demonstrated in the scatter plot, Pc AHDBS and Pc SCSN are the most symmetric karyotypes in terms of intra- and inter-chromosomal index, respectively, while Pk YNKM is the most asymmetric karyotype in terms of both intra- and inter-chromosomal asymmetry.

**Figure 4.**
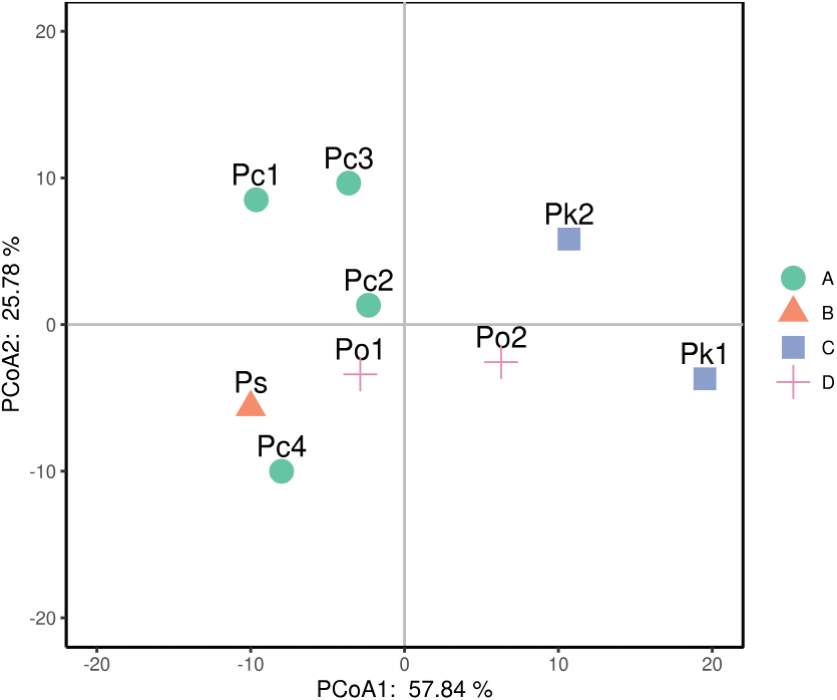
PCoA for the 9 populations of four *Polygonatum* species, *P.cyrtonema* (Pc), *P.kingianum* (Pk), *P.odoratum* (Po) and *P.sibiricum* (Ps), based on *x*, 2*n*, TCL, M_CA_, CV_CL_ and CV_CI_. Pc1, Pc2, Pc3 and Pc4 represent Pc AHDBS, Pc HNHH, Pc HBHS and Pc SCSN, respectively. Pk1 and Pk2 represent Pk YNKM and Pk YNWS, respectively. Po1 and Po2 represent Po HNXH and Po AHDBS, respectively. Ps represents Ps HNFNS. PCoA1 reflects the original data characteristics before the dimensionality reduction of 57.84%. PCoA2 reflects the character of the original data before the dimensionality reduction of 25.78%. The sum of the two percentages is 83.62%, indicating that the two-dimensional coordinate system can reflect the characteristics of 83.62% of the original data.

PCoA based on the six quantitative karyological parameters reveals the karyological relationships among the 9 populations (Fig. 4). The PCoA scatter plot shows that the 9 populations are divided into two groups along the direction of PCoA1: Pc AHDBS, Pc HNHH, Pc HBHS, Pc SCSN, Po HNXH and Ps HNFNS in one group with closely clustering together of Pc AHDBS and Pc HBHS , Pc HNHH and Po HNXH, Pc SCSN and Ps HNFNS, respectively; while Po AHDBS, Pk YNKM and Pk YNWS in another group with Pk YNKM occupying the most isolated position. Po HNXH and Po AHDBS occupy on either side of the middle position and are close to each other along the direction of PCoA2.

### ﻿FISH mapping of 5S and 45S rDNA sites

The FISH results show inter- and intra-specific variations in number and location of 5S and 45S rDNA loci (Figs 1, 2; Table 2). All but two populations have a single locus of 5S rDNA, which in Pc AHDBS, Pc HNHH, Pc SCSN, Po HNXH and Ps HNFNS is situated in the distal or interstitial regions of the long arms of a small m or sm chromosome pair (Figs 1A, B, D, G, I, 2A, B, D, G, I; Table 2). Pc HBHS and Po AHDBS have one 5S locus in the same position as the five populations described above and another 5S locus that is located in the proximal regions of the long arms of a large st or sm chromosome pair (in Po AHDBS only one member of the chromosome pair showed 5S rDNA signal) (Figs 1C, H, 2C, H; Table 2). The single 5S locus in Pk YNKM and Pk YNWS is located in the proximal regions of the short arms of a large st chromosome pair (Figs 1E, F, 2E, F; Table 2).

**Table 2.** The number (pair) and location of rDNA loci in 9 populations of four *Polygonatum* species.

Populations^†^	5S rDNA^‡^	45S rDNA^‡^
Pc AHDBS	one[8L-DIS(78.73%)]	two[3L-INT^§^(57.57%),6L-INT^§^(65.28%)]
Pc HNHH	one[10L-INT(61.65%)]	two[1L-INT(49.19%)^§^,4L-INT(57.46%)^§^]
Pc HBHS	two[2L-PRO(19.28%),10L-DIS(79.08%)]	two[1L-INT(45.14%)^§^,4L-INT(59.20%)^§^]
Pc SCSN	one[9L-DIS(78.08%)]	three[3S-INT(44.35%)^§^, 8S-DIS(82.02%)^§^, 10L-INT(46.75%)]
Pk YNKM	one[1S-PRO(23.36%)]	three[4L-INT(61.57%)^§^,7L-INT(65.24%)^§^, 8L-INT(70.65%)^§^]
Pk YNWS	one[2S-PRO(21.29%)]	two[3L-INT(44.50%)^§^, 8L-INT(70.88%)^§^]
Po HNXH	one[9L-INT(58.06%)]	two[2L-INT(57.05%)^§^, 5L-INT(65.56%)^§^]
Po AHDBS	one and a half [1L-PRO(18.29%)^|^ , 10L-DIS(82.99%)]	two[2L-INT(65.41%)^§^, 5L-INT(54.92%)^§^]
Ps HNFNS	one[11L-INT(71.79%)]	two[1S-INT(33.76%)^§^, 3L-INT(71.86%)^§^]

^†^Pc = P.cyrtonema, Pk = P.kingianum, Po = P.odoratum, Ps = P.sibiricum. ^‡^S and L represent the short and long arms, respectively; CEN, PRO, INT, DIS and TER represent the centromeric (*di* = 0), proximal (0 < *di* < 25%), interstitial (25% ≤ *di* ≤ 75%), distal (75% < *di* < 100%) and terminal (*di* = 100%) positions, respectively; figures ahead of the positions designate the chromosomal pair involved; the percentages in square brackets are the percentage distance from centromere to rDNA locus (*di* = *d* × 100/*a*; *d* = distance of the center of FISH signals from the centromere, *a* = length of the corresponding chromosome arm). ^§^indicates the 45S rDNA loci generating secondary constrictions (SCs). ^|^ only a member of the chromosome pair has 5S rDNA site.

As for 45S rDNA site, there exist two loci in Pc AHDBS, Pc HNHH, Pc HBHS, Pk YNWS, Po HNXH, Po AHDBS and Ps HNFNS and three loci in Pc SCSN and Pk YNKM (Figs 1, 2; Table 2). All but one 45S loci in the 9 populations generated SCs (Fig. 2, Suppl. material 3: fig. S1). No SC was observed within the 45S locus that was located on the long arms of pair 10 of Pc SCSN (Fig. 2D, Suppl. material 3: fig. S1D). Among the four populations of *P.cyrtonema*, the locations of the two 45S loci of Pc HNHH and Pc HBHS are almost the same, being located in interstitial regions of the long arms of pairs 1 and 4 (Fig. 2B, C; Table 2); in Pc AHDBS, the lengths and arm ratios of the chromosome pairs with the two 45S loci are changed compared to Pc HNHH and Pc HBHS, but both loci are still located in interstitial regions of the long arms of two large chromosome pairs (the 3^rd^ and 6^th^ pairs) (Fig. 2A; Table 2; Suppl. material 2: table S2); in Pc SCSN, the two 45S loci which generate SCs are located on the short arms of pairs 4 and 7 and a new minor 45S locus appear in pair 10 (Fig. 2D; Table 2). Among the two populations of *P.kingianum*, the 45S locus that is located on the long arms of pair 8 is conserved, the location of the 45S locus that is located on the long arms of a large st chromosome pair (the 4^th^ or 6^th^ pair) differs somewhat (being closer to the distal region in Pk YNWS than in Pk YNKM), and an additional 45S locus appears on the long arms of pair 7 in Pk YNKM (Fig. 2E, F; Table 2). In *P.odoratum*, the two 45S loci are located on the long arms of pairs 2 and 5 with similar percentage distance between Po HNXH and Po AHDBS, but the arm ratios of the chromosome pairs with the respective loci differ significantly between the two populations (Fig. 2G, H; Table 2; Suppl. material 2: table S2). In *P.sibiricum*, the two 45S loci are located on the short arms of a large chromosome pair (the 1^st^ pair) and the long arms of another large chromosome pair (the 3^rd^ pair), being different from all other populations with two 45S loci (Fig. 2I; Table 2).

## ﻿Discussion

### ﻿Karyotype variation

In the present study, a primary molecular cytogenetic characterization of 9 populations of *P.cyrtonema*, *P.kingianum*, *P.odoratum* and *P.sibiricum* is conducted for the first time. The karyotypic parameters and rDNA patterns vary among the populations studied, enabling an accurate distinguishment between individual genomes. The rDNA FISH signals provide new chromosomal markers for investigating the inter- and intra-specific karyotype evolution in the genus *Polygonatum*.

The evolution of chromosome number in *Polygonatum* is mainly dysploidy, and a few species have polyploidy (Deng et al. 2009; Zhao et al. 2014; Wang et al. 2016a). There are two levels of dysploidy in *Polygonatum*. First, there is a significant variation of basic chromosome number among different species, from *x* = 8 to *x* = 16 (Deng et al. 2009; Zhao et al. 2014; Wang et al. 2016a; Zhou et al. 2020). On the other hand, there exists also intraspecific dysploid variation in more than fourteen species in which *P.cyrtonema*, *P.kingianum*, *P.odoratum* and *P.sibiricum* are involved (Zhao et al. 2014; Wang et al. 2016a; Zhou et al. 2020). Moreover, a few species such as *P.cyrtonema* and *P.odoratum* display continuous dysploidy (Wang et al. 2016a). We analyze the basic chromosome numbers of all diploid populations of each of the four species including the populations in our study and those reported in the literature, and calculate the frequency of occurrence of each basic chromosome number in each species (Suppl. material 4: fig. S2). We infer that, in each species, the basic chromosome number that occurs most frequently should be the original character and other basic chromosome numbers should be derived character. Previous studies reported *x* = 9, 10, 11 and 12 for *P.cyrtonema* (Fang et al. 1984; Wang et al. 1987, 1991; Chen et al. 1989; Tamura 1990; Shao et al. 1993; Wu et al. 2001; Jin et al. 2002; Chen and Zhou 2005; Zhao et al. 2014; Zhou et al. 2020), a species occurs in south, southeast and southwest China (Chen and Tamura 2000). Our study detected a new basic chromosome number (*x* = 13) for *P.cyrtonema*, further demonstrating the existence of continuous dysploidy within this species. Among the five basic chromosome numbers of *P.cyrtonema*, *x* = 11 occurs most frequently (accounting for 60.98%; Suppl. material 4: fig. S2A). For *P.kingianum*, a species occurs in Sichuan, Yunnan and Guizhou provinces, China (Chen and Tamura 2000), *x* = 13 and 15 have been reported, which were the basic chromosome numbers of the populations from Sichuan and Yunnan, respectively (Yang et al. 1988; Chen et al. 1989; Tamura 1993; Wang et al. 1993; Deng et al. 2009; Zhou et al. 2020). The chromosome numbers (2*n* = 30) of the two *P.kingianum* populations from Yunnan that we analyzed here are consistent with those of the Yunnan populations reported previously (Tamura 1993; Wang et al. 1993; Zhou et al. 2020). The basic chromosome number of wide-ranging Eurasian species *P.odoratum* has been reported as *x* = 8, 9, 10 and 11 (Li et al. 1980; Wang et al. 1987, 1988; Chen 1989; Fang 1989; Fu and Hong 1989; Tamura 1990; Wang et al. 1991; Shang et al. 1992; Shao et al. 1993; Han et al. 1998; Wu et al. 2001; Weiss-Schneeweiss and Jang 2003; Chen and Zhou 2005; Zhao et al. 2014; Wang et al. 2016a; Zhou et al. 2020) with *x* = 10 occurring most frequently (accounting for 75.00%; Suppl. material 4: fig. S2C). Two of the four basic numbers (*x* = 10 and 11) are detected in the *P.odoratum* populations studied here. It has been showed that most populations of *P.odoratum* from Europe, northeast Asia, northwest and north China had a chromosome number of 2*n* = 20, while populations from east, central and southwest China had a chromosome number fluctuated around 2*n* = 20 (Fang 1989). For *P.sibiricum*, a species occurs in northeastern, northern, central and eastern China, Korea, Mongolia and Russia (Siberia) (Chen and Tamura 2000), *x* = 12, 15 and 18 has been reported (Mehra and Pathania 1960; Mehra and Sachdeva 1976; Fang et al. 1984; Wang et al. 1987; Chen 1989; Han et al. 1998; Deng et al. 2009; Zhao et al. 2014; Zhou et al. 2020). The *P.sibiricum* population used in this study (Ps HNFNS) shows the basic chromosome number (*x* = 12) that occurs most frequently in this species (accounting for 85.71%; Suppl. material 4: fig. S2D).

The scatter plot of M_CA_ vs. CV_CL_ reveals that the karyotypic structures vary both among species and among different populations of the same species in terms of both intra- and inter-chromosomal asymmetry (Fig. 3). There are significant variations in the chromosomal organization of the complements between populations with different basic chromosome numbers of the same species. In *P.cyrtonema*, the karyotypes of the populations with *x* = 9, 10, 11 and 12 are usually of distinct bimodality, whose number of large and small chromosomes are 6 + 3 (Fang et al. 1984; Shao et al. 1993; Zhao et al. 2014; Zhou et al. 2020; this study), 5 + 5 or 6 + 4 (Chen 1989; Wang et al. 1991; Shao et al. 1993; Zhou et al. 2020), 5 + 6 (Wang et al. 1987, 1991; Shao et al. 1993; Jin et al. 2002; Chen and Zhou 2005; Zhou et al. 2020; this study) and 4 + 8 (Jin et al. 2002), respectively. However, the karyotype of the population with *x* = 13 becomes indistinctly bimodal (Fig. 2D; Suppl. material 2: table S2). Similarly, the karyotypes of the *P.odoratum* populations with *x* = 8, 9, 10 and 11 are mainly of distinct bimodality, whose number of large and small chromosomes are 6 + 2 (Shao et al. 1993), 7 + 2 or 6 + 3 (Wang et al. 1988; Shao et al. 1993; Wu et al. 2001; Chen and Zhou 2005; Zhao et al. 2014), 6 + 4 or 7 + 3 (Chen 1989; Tamura 1990; Wu et al. 2001; Weiss-Schneeweiss and Jang 2003; Zhao et al. 2014; Zhou et al. 2020; this study) and 5 + 6 (Fang 1989; Fu and Hong 1989; Zhao et al. 2014; this study), respectively. Some of the *P.odoratum* populations with *x* = 10 reported previously had unimodal karyotypes (Fang 1989; Hong and Zhu 1990; Tamura 1990, 1993; Wang et al. 1991; Shang et al. 1992; Han et al. 1998). As mentioned above, *x* = 11 and 10 should be the original basic chromosome numbers of *P.cyrtonema* and *P.odoratum*, respectively, thus there should be a concomitant decrease and increase of basic chromosome number on the basis of *x* = 11 or 10 in the continuous dysploid variation of the two species. Compared with Pc HNHH and Pc HBHS (*x* = 11), Pc AHDBS (*x* = 9) increased by one pair of large chromosomes and decreased by three pairs of small chromosomes with a production of two pairs of large m chromosomes (the 1^st^ and 3^rd^ pairs) and changes of the relative lengths and arm ratios of the chromosomes bearing 45S rDNA loci (Fig. 2A, B, C), and Pc SCSN increased by two pairs of chromosomes, and underwent translocations of the two major 45S loci from long arms to short arms and loss of bimodality (Fig. 2D). Compared with Po HNXH (*x* = 10), Po AHDBS (*x* = 11) increased by two pairs of small chromosomes (probably the 6^th^ and 8^th^ pairs), underwent changes of the arm ratios of the chromosomes bearing the two 45S loci as well as the percentage distances of both 45S and 5S loci (Fig. 2G, H). The above analysis of intraspecific increase and decrease of basic chromosome number in *P.cyrtonema* and *P.odoratum* shows that there exists basically a one-to-two or two-to-one relationship between changes in the number of large and small chromosomes in the continuous dysploid variation, but there are not any small st and t chromosomes in their karyotypes (Fig. 2A, B, C, G, H). Thus, the intraspecific dysploidy was not a classic Robertsonian transformation (chromosomal fission or fusion) process (Olson and Gorelick 2011). Considering that changes of the relative lengths and arm ratios of some chromosomes are accompanied, and even the bimodality of karyotypes of some populations has been lost, we suggest that complex chromosomal rearrangements, probably including centromere fission or fusion, unequal translocations, and pericentric inversions, have contributed to the continuous dysploid variation within these species (Moscone et al. 2007).

Chromosome arrangements also occur between populations with the same basic chromosome number. The karyotypes of Pc HNHH and Pc HBHS (both *x* = 11) show some differences, mainly including significant changes of the arm ratios of pairs 2 and 8 between the two populations, and the occurrence of another 5S locus on pair 2 in Pc HBHS (Fig. 2B, C; Suppl. material 2: table S2). Among the reported populations of *P.cyrtonema* with *x* = 10, the majority had bimodal karyotypes composed of 5 + 5 (Chen et al. 1989; Shao et al. 1993) or 6+4 (Wang et al. 1991; Zhou et al. 2020), a few had unimodal karyotypes (Wang et al. 1991), indicating multiple chromosomal arrangements between the populations with *x* = 10. Also, the karyotypes of Pk YNKM and Pk YNWS (both *x* = 15) have some differences, mainly including significant changes in the arm ratios of pairs 5 and 9 between the two populations, changes in the percentage distance of their sharing two pairs of 45S loci, and the presence of an additional 45S locus on pair 7 of Pk YNKM (Fig. 2E, F; Suppl. material 2: table S2). As for *P.odoratum*, previous reports showed that some populations with *x* = 10 had bimodal karyotypes composed of 7 + 3 (Shang et al. 1992; Wu et al. 2001) or even unimodal karyotype (Wang et al. 1987, 1988, 1991; Fang 1989; Hong and Zhu 1990; Tamura 1990, 1993; Han et al. 1998) instead of a bimodal karyotype composed of 6 + 4 as the populations studied by us and other previous authors (Chen 1989; Weiss-Schneeweiss and Jang 2003; Zhao et al. 2014; Zhou et al. 2020), indicating the occurrence of multiple chromosome rearrangements among different populations with *x* = 10.

Although the chromosomal rearrangements inferred from the changes in chromosomal morphology and rDNA pattern may only represent the tip of the iceberg of the dysploidy within species of the genus *Polygonatum*. However, it has been revealed that, in the evolutionary process, geographically diverse populations of *Polygonatum* species are easy to preserve large-scale and multiple chromosomal rearrangements. The reasons for this may be the perennial and clonal nature of *Polygonatum* species (Wang et al. 1987). It is the abundant chromosomal rearrangements and the resulting dysploid variation that leads to the highly morphological variation within widely-distributed *Polygonatum* species such as *P.cyrtonema* and *P.odoratum* (Wang et al. 1991; Shao et al. 1993).

The direction of the basic chromosome number evolution in the interspecific dysploidy of *Polygonatum* has long been an important and challenging issue in the cytogenetic study of the genus. From our comparative molecular cytogenetic karyotype analysis, there are obvious differences in chromosome number, karyotypic structure and rDNA pattern among *P.cyrtonema* and *P.odoratum* (representatives of sect. Polygonatum), *P.kingianum* (a representative of sect. Verticillata) and *P.sibiricum* (the representative of sect. Sibirica) (Meng et al. 2014). It is generally believed that, in morphology, the section with alternate phyllotaxy is relatively primitive and the section with whorled (verticillate) phyllotaxy is relatively evolved (Wang et al. 1987; Shao et al. 1994). Therefore, it was speculated that ascending dysploidy may be the main evolutionary mode of the karyotype in *Polygonatum* (Deng et al. 2009). Wang et al. (1987) speculated that the ancestral basic chromosome number of *Polygonatum* was most likely *x* = 10, based on which the ascending dysploidy was predominant and the descending dysploidy was secondary. Bayesian analyses of the molecular phylogenetic study based on four regions of chloroplast genomes supported the alternate-leaf arrangement as the ancestral state for *Polygonatum* (Meng et al. 2014). However, a recent comparative analysis of chloroplast genomes of *Polygonatum* species showed that the verticillate leaf might be the ancestral state of this genus (Wang et al. 2022). Therefore, further studies are needed to determine whether the interspecific dysploidy of *Polygonatum* is ascending or descending.

### ﻿Phylogenetic relationships

According to the infrageneric classification system of Meng et al. (2014) and recent molecular phylogenetic studies of *Polygonatum* (Wang et al. 2016b; Floden and Schilling 2018; Zhao et al. 2019; Wang et al. 2022; Xia et al. 2022), *P.cyrtonema* and *P.odoratum* are placed on different sister branches of the same lineage of sect. Polygonatum, *P.sibiricum*, the only species of sect. Sibirica, is sister to sect. Polygonatum in one major branch, and *P.kingianum* is placed in another major branch (sect. Verticillata). Comparison of the karyotypic structures and rDNA patterns of these four representative species is helpful to reveal the chromosome evolution among three sections of the genus and the phylogenetic relationships among these species. However, this comparison is complicated by the presence of dysploid variation within these species which results in intra-specific variations in both karyotypic structure and rDNA pattern. As mentioned above, the original basic chromosome numbers of *P.cyrtonema*, *P.kingianum*, *P.odoratum* and *P.sibiricum* should be *x* = 11, 15, 10 and 12, respectively (Suppl. material 4: fig. S2), so it is both reasonable and valid to use the populations with these basic chromosome numbers (Pc HNHH, Pc HBHS, Pk YNKM, Pk YNWS, Po HNXH and Ps HNFNS) for comparison.

The similarities and differences in rDNA patterns among species reflect the closeness of relatedness between species (e.g. Moscone et al. 2007; Chacón et al. 2012; Siljak-Yakovlev and Peruzzi 2012; She et al. 2015, 2020; Senderowicz et al. 2022; Yucel et al. 2022; Mitrenina et al. 2023). Among the 9 populations of the four species investigated here, seven populations have one 5S rDNA locus and two 45S rDNA loci, suggesting one locus of 5S rDNA and two loci of 45S rDNA being the ancestral state of *Polygonatum* species. Another 5S locus in Pc HBHS (localization: 2L-PRO), a half locus of 5S in Po AHDBS (localization: 1L-PRO), another 45S locus in Pc SCSN (localization: 10L-INT) and Pk YNKM (localization: 7L-INT) probably originated from chromosomal arrangements (Table 2; Fig. 2C, D, E, H) (Chacón et al. 2012; She et al. 2015; Senderowicz et al. 2022) or the action of transposable elements which accumulate at the proximity or around rDNA loci (Raskina et al. 2008). As for the distribution of the conserved locus of 5S rDNA, that of *P.cyrtonema* (Pc HNHH and Pc HBHS), *P.odoratum* (Po HNXH) and *P.sibiricum* (Ps HNFNS) is located in the interstitial or distal regions of the long arms of a pair of small m or sm chromosomes, while that of *P.kingianum* (Pk YNKM and Pk YNWS) is located in the proximal regions of the short arms of a pair of large st chromosomes (Fig. 2B, C, E, F, G, I). With regard to the distribution of the two conserved loci of 45S rDNA, those of *P.cyrtonema* (Pc HNHH and Pc HBHS) and *P.odoratum* (Po HNXH) are located in the interstitial regions of the long arms of two pairs of large chromosomes; those of *P.sibiricum* (Ps HNFNS) are also located on two pairs of large chromosomes, but one is located in the interstitial regions of the short arms and the other in the interstitial regions of the long arms; one of those of *P.kingianum* is located in the interstitial regions of the long arms of a pair of large chromosomes, and the other in the interstitial regions of the long arms of a pair of small chromosome (Fig. 2B, C, E, F, G, I). These facts suggest that, among the four species, *P.cyrtonema* and *P.odoratum* are most closely related to each other, and *P.cyrtonema* and *P.odoratum* are closely related to *P.sibiricum* and distantly related to *P.kingianum*. This inference is consistent with the phylogenetic relationships among these species revealed by molecular phylogenetic studies (Meng et al. 2014; Wang et al. 2016b; Floden and Schilling 2018; Zhao et al. 2019; Wang et al. 2022; Xia et al. 2022).

PCoA based on *x*, 2*n*, TCL, CV_CI_, M_CA_, CV_CL_ is – thus far – the most legitimate approach to use for comparing karyotypes and reconstructing karyological relationships among taxa (Peruzzi and Altınordu 2014; Dehery et al. 2020; Kadluczka and Grzebelus 2021; She et al. 2023). It seems that the karyological relationships between the four species are not clearly delineated by the PCoA scatter plot of the 9 populations since Pc SCSN and Ps HNFNS are closely clustered, and Po AHDBS is distantly separated from Po HNXH and placed in the group that *P.kingianum* is in (Fig. 4). However, when only the populations with the original basic chromosome numbers are considered, the karyological relationships among the four species are basically consistent with the molecular phylogenetic relationships among these species (Meng et al. 2014; Wang et al. 2016b; Floden and Schilling 2018; Zhao et al. 2019; Wang et al. 2022; Xia et al. 2022). As the PCoA scatter plot showed (Fig. 4), along the direction of PCoA1, *P.cyrtonema* (Pc HNHH and Pc HBHS), *P.odoratum* (Po HNXH) and *P.sibiricum* (Ps HNFNS) are in one group with the former two species (Pc HNHH and Po HNXH) closely clustering, while *P.kingianum* (Pk YNKM and Pk YNWS) was in another group and away from the middle position of the two groups. Therefore, it is effective to use populations with the original basic chromosome number of each species for PCoA-based karyological relationship construction among species that possess intraspecific dysploidy.

## ﻿Conclusions

Detailed molecular cytogenetic karyotypes of 9 populations of four *Polygonatum* species, *P.cyrtonema*, *P.kingianum*, *P.odoratum* and *P.sibiricum*, are established for the first time using the dataset of chromosome measurements and FISH signals of 5S and 45S rDNA. Comparative karyotyping reveals distinct variations in the karyotypic parameters and rDNA patterns among and within species, and intraspecific dysploidy of *P.cyrtonema* and *P.odoratum*. The evolutionary relationships among the four species revealed by rDNA pattern comparison and PCoA based on *x*, 2*n*, TCL, CV_CI_, M_CA_ and CV_CL_ are basically accordant with the phylogenetic relationships revealed by molecular phylogenetic studies.

## Supplementary materials

Supplementary material 1 The plant materials Data type doc https://binary.pensoft.net/file/1050427This dataset is made available under the Open Database License (http://opendatacommons.org/licenses/odbl/1.0/). The Open Database License (ODbL) is a license agreement intended to allow users to freely share, modify, and use this Dataset while maintaining this same freedom for others, provided that the original source and author(s) are credited.Yan-Fang Wei, Xiang-Hui Jiang, Rong Song, Chao-Wen She

Supplementary material 2 Chromosome measurements of 9 populations of four *Polygonatum* species Data type docx https://binary.pensoft.net/file/1050428This dataset is made available under the Open Database License (http://opendatacommons.org/licenses/odbl/1.0/). The Open Database License (ODbL) is a license agreement intended to allow users to freely share, modify, and use this Dataset while maintaining this same freedom for others, provided that the original source and author(s) are credited.Yan-Fang Wei, Xiang-Hui Jiang, Rong Song, Chao-Wen She

Supplementary material 3 Metaphase chromosomes of 9 populations of four *Polygonatum* species, *P.cyrtonema* (Pc), *P.kingianum* (Pk), *P.odoratum* (Po) and *P.sibiricum* (Ps) Data type docx https://binary.pensoft.net/file/1050429This dataset is made available under the Open Database License (http://opendatacommons.org/licenses/odbl/1.0/). The Open Database License (ODbL) is a license agreement intended to allow users to freely share, modify, and use this Dataset while maintaining this same freedom for others, provided that the original source and author(s) are credited.Yan-Fang Wei, Xiang-Hui Jiang, Rong Song, Chao-Wen She

Supplementary material 4 Diagrams of the distribution of basic chromosome numbers within four *Polygonatum* species, *P.cyrtonema*, *P.kingianum*, *P.odoratum* and *P.sibiricum* Data type docx https://binary.pensoft.net/file/1050430This dataset is made available under the Open Database License (http://opendatacommons.org/licenses/odbl/1.0/). The Open Database License (ODbL) is a license agreement intended to allow users to freely share, modify, and use this Dataset while maintaining this same freedom for others, provided that the original source and author(s) are credited.Yan-Fang Wei, Xiang-Hui Jiang, Rong Song, Chao-Wen She
