# Supplementary material for: ﻿Molecular cytogenetic characterization of 9 populations of four species in the genus Polygonatum (Asparagaceae)
Source: Comp Cytogenet. 2024 May 16;18:73–95. doi: 10.3897/compcytogen.18.122399 (PMC11116888; doi:10.3897/compcytogen.18.122399)
Supplement: Supplementary material 1 — The plant materials [file comparative_cytogenetics-18-073_article-122399__-s001.doc]

**Table S1. The plant materials**

| Populations | Species | Locality |
| --- | --- | --- |
| Pc AHDBS | *Polygonatum cyrtonema* | Dabieshan, Anhui, China |
| Pc HNHH | *Polygonatum cyrtonema* | Huaihua, Hunan, China |
| Pc HBHS | *Polygonatum cyrtonema* | Huangshi, Hubei, China |
| Pc SCSN | *Polygonatum cyrtonema* | Suining, Shichun, China |
| Pk YNKM | *Polygonatum kingianum* | Kunming, Yunan, China |
| Pk YNWS | *Polygonatum kingianum* | Wenshan, Yunan, China |
| Po HNXH | *Polygonatum odoratum* | Xinhua, Hunan, China |
| Po AHDBS | *Polygonatum odoratum* | Dabieshan, Anhui, China |
| Ps HNFNS | *Polygonatum sibiricum* | Funiushan, Henan, China |
