# Supplementary material for: ﻿Molecular cytogenetic characterization of 9 populations of four species in the genus Polygonatum (Asparagaceae)
Source: Comp Cytogenet. 2024 May 16;18:73–95. doi: 10.3897/compcytogen.18.122399 (PMC11116888; doi:10.3897/compcytogen.18.122399)
Supplement: Supplementary material 2 — Chromosome measurements of 9 populations of four Polygonatum species [file comparative_cytogenetics-18-073_article-122399__-s002.docx]

**Table S2. Chromosome measurements of 9** **populations of four *Polygonatum* species.**

| Population^※^ | Chr. No. | Relative length (%) | | | Arm ratio  ± SD | Type |
| --- | --- | --- | --- | --- | --- | --- |
|  |  | Short arm  ± SD | Long arm  ± SD | Total  ± SD |  |  |
|  |  |  |  |  |  |  |
| Pc AHDBS | 1 | 6.78 ± 0.23 | 10.23 ± 0.01 | 17.01 ± 0.22 | 1.51 ± 0.05 | m |
|  | 2 | 3.79 ± 0.07 | 9.95 ± 0.24 | 13.74 ± 0.27 | 2.62 ± 0.07 | sm |
|  | 3 | 5.49 ± 0.16 | 7.95 ± 0.24 | 13.44 ± 0.34 | 1.45 ± 0.04 | m^†^ |
|  | 4 | 3.60 ± 0.40 | 8.97 ± 0.64 | 12.56 ± 0.33 | 2.53 ± 0.45 | sm |
|  | 5 | 3.14 ± 0.17 | 9.12 ± 0.40 | 12.26 ± 0.33 | 2.91 ± 0.26 | sm |
|  | 6 | 2.70 ± 0.16 | 8.52 ± 0.31 | 11.22 ± 0.15 | 3.16 ± 0.30 | st^†^ |
|  | 7 | 2.88 ± 0.13 | 4.39 ± 0.14 | 7.27 ± 0.18 | 1.53 ± 0.09 | m |
|  | 8 | 2.51 ± 0.11 | 3.77 ± 0.20 | 6.27 ± 0.25 | 1.50 ± 0.10 | m |
|  | 9 | 2.64 ± 0.04 | 3.60 ± 0.38 | 6.23 ± 0.39 | 1.36 ± 0.14 | m |
|  | Total | 33.52 ± 1.47 | 66.48 ± 2.56 | 100 |  |  |
| Pc HNHH | 1 | 3.86 ± 0.18 | 9.20 ± 0.71 | 13.06 ± 0.77 | 2.39 ± 0.19 | sm^†^ |
|  | 2 | 3.57 ± 0.38 | 9.10 ± 0.34 | 12.68 ± 0.11 | 2.57 ± 0.35 | sm |
|  | 3 | 3.48 ± 0.12 | 8.79 ± 0.27 | 12.28 ± 0.37 | 2.53 ± 0.07 | sm |
|  | 4 | 3.05 ± 0.42 | 8.90 ± 0.87 | 11.95 ± 0.91 | 2.96 ± 0.50 | sm^†^ |
|  | 5 | 2.31 ± 0.26 | 9.24 ± 1.13 | 11.56 ± 0.97 | 4.06 ± 0.90 | st |
|  | 6 | 2.70 ± 0.10 | 4.96 ± 0.26 | 7.66 ± 0.30 | 1.84 ± 0.10 | sm |
|  | 7 | 2.85 ± 0.25 | 4.01 ± 0.44 | 6.86 ± 0.61 | 1.41 ± 0.13 | m |
|  | 8 | 1.68 ± 0.25 | 4.64 ± 0.46 | 6.32 ± 0.48 | 2.81 ± 0.56 | sm |
|  | 9 | 2.72 ± 0.24 | 3.38± 0.11 | 6.10 ± 0.32 | 1.25 ± 0.09 | m |
|  | 10 | 2.26 ± 0.35 | 3.81 ± 0.40 | 6.07 ± 0.59 | 1.71 ± 0.31 | sm |
|  | 11 | 1.83 ± 0.55 | 3.63 ± 0.65 | 5.46 ± 0.40 | 2.17 ± 0.90 | sm |
|  | Total | 30.31 ± 3.12 | 69.65 ± 5.65 | 100 |  |  |
| Pc HBHS | 1 | 4.23 ± 0.53 | 8.85 ± 0.62 | 13.08 ± 0.19 | 2.13 ± 0.44 | sm^†^ |
|  | 2 | 2.74 ± 0.17 | 9.88 ± 0.39 | 12.62 ± 0.49 | 3.61 ± 0.19 | st |
|  | 3 | 3.52 ± 0.15 | 9.46 ± 0.15 | 12.98 ± 0.21 | 2.69 ± 0.12 | sm |
|  | 4 | 3.10 ± 0.24 | 9.60 ± 0.96 | 12.70 ± 0.85 | 3.12 ± 0.48 | st^†^ |
|  | 5 | 2.30 ± 0.44 | 9.72 ± 0.62 | 12.02 ± 0.19 | 4.36 ± 1.00 | st |
|  | 6 | 2.58 ± 0.76 | 4.45 ± 0.46 | 7.03 ± 0.34 | 1.90 ± 0.88 | sm |
|  | 7 | 2.78 ± 0.17 | 3.93 ± 0.28 | 6.71 ± 0.24 | 1.42 ± 0.17 | m |
|  | 8 | 2.40 ± 0.74 | 3.88 ± 0.81 | 6.28 ± 0.11 | 1.83 ± 1.05 | sm |
|  | 9 | 2.11 ± 0.16 | 3.54 ± 0.20 | 5.65 ± 0.19 | 1.68 ± 0.18 | m |
|  | 10 | 2.64 ± 0.18 | 3.27 ± 0.31 | 5.91 ± 0.46 | 1.24 ± 0.07 | m |
|  | 11 | 1.66 ± 0.02 | 3.36 ± 0.40 | 5.01 ± 0.39 | 2.02 ± 0.25 | sm |
|  | total | 30.06 ± 4.54 | 69.94 ± 5.11 | 100 |  |  |
| Pc SCSN | 1 | 2.97 ± 0.22 | 7.43 ± 0.53 | 10.41 ± 0.61 | 2.51 ± 0.23 | sm |
|  | 2 | 2.26 ± 0.21 | 7.55 ± 0.11 | 9.81± 0.11 | 3.37 ± 0.36 | st |
|  | 3 | 3.19 ± 0.40 | 6.58 ± 0.40 | 9.77 ± 0.50 | 2.08 ± 0.30 | sm^†^ |
|  | 4 | 1.82 ± 0.17 | 6.81 ± 0.38 | 8.63 ± 0.54 | 3.74 ± 0.13 | st |
|  | 5 | 2.73 ± 0.15 | 5.43 ± 0.32 | 8.16 ± 0.45 | 1.99 ± 0.06 | sm |
|  | 6 | 3.08 ± 0.18 | 5.07 ± 0.13 | 8.15 ± 0.19 | 1.65 ± 0.12 | m |
|  | 7 | 2.71 ± 0.33 | 5.41 ± 0.20 | 8.12 ± 0.46 | 2.02 ± 0.21 | sm |
|  | 8 | 2.03 ± 0.44 | 5.80 ± 0.10 | 7.83 ± 0.36 | 2.97 ± 0.79 | sm^†^ |
|  | 9 | 2.34 ± 0.27 | 3.86 ± 0.09 | 6.20 ± 0.36 | 1.66 ± 0.16 | m |
|  | 10 | 2.43 ± 0.32 | 3.62 ± 0.04 | 6.05 ± 0.33 | 1.50 ± 0.21 | m |
|  | 11 | 1.76 ± 0.13 | 4.21 ± 0.33 | 5.97 ± 0.38 | 2.40 ± 0.24 | sm |
|  | 12 | 2.28 ± 0.75 | 3.41 ± 0.39 | 5.69 ± 0.83 | 1.59 ± 0.47 | m |
|  | 13 | 1.85 ± 0.08 | 3.37 ± 0.31 | 5.21 ± 0.35 | 1.82 ± 0.15 | sm |
|  | Total | 31.46 ± 3.64 | 68.54 ± 3.33 | 100 |  |  |
| Pk YNKM | 1 | 2.20 ± 0.12 | 8.37 ± 0.14 | 10.57 ± 0.23 | 3.82 ± 0.16 | st |
|  | 2 | 1.96 ± 0.37 | 8.53 ± 0.30 | 10.49 ± 0.59 | 4.44 ± 0.77 | st |
|  | 3 | 2.38 ±1.14 | 8.05 ± 0.68 | 10.43 ± 0.81 | 3.95 ±1.85 | st |
|  | 4 | 2.14 ± 0.20 | 7.99 ± 0.20 | 10.13 ± 0.40 | 3.75 ± 0.26 | st^†^ |
|  | 5 | 1.45 ± 0.13 | 7.46 ± 1.05 | 8.91 ± 0.94 | 5.23 ± 1.16 | st |
|  | 6 | 1.49 ± 0.10 | 6.33 ± 0.79 | 7.82 ± 0.89 | 4.24 ± 0.25 | st |
|  | 7 | 1.22 ± 0.11 | 5.86 ± 0.08 | 7.08 ± 0.04 | 4.84 ± 0.46 | st^†^ |
|  | 8 | 1.94 ± 0.14 | 4.49 ± 0.59 | 6.43 ± 0.73 | 2.31 ± 0.14 | sm^†^ |
|  | 9 | 1.59 ± 0.15 | 4.04 ± 1.10 | 5.64 ± 1.19 | 2.53 ± 0.56 | sm |
|  | 10 | 1.32 ± 0.10 | 3.12 ± 0.21 | 4.43 ± 0.16 | 2.38 ± 0.32 | sm |
|  | 11 | 1.52 ± 0.21 | 2.85 ± 0.29 | 4.37 ± 0.09 | 1.91 ± 0.42 | sm |
|  | 12 | 1.47 ± 0.14 | 2.55 ± 0.61 | 4.02 ± 0.49 | 1.76 ± 0.54 | sm |
|  | 13 | 1.54 ± 0.09 | 1.88 ± 0.24 | 3.42 ± 0.33 | 1.22 ±0.08 | m |
|  | 14 | 1.30 ± 0.05 | 2.12 ± 0.27 | 3.42 ± 0.25 | 1.63 ± 0.24 | m |
|  | 15 | 1.20 ± 0.10 | 1.65 ± 0.14 | 2.85 ± 0.24 | 1.38 ± 0.03 | m |
|  | Total | 24.72 ± 3.15 | 75.28 ± 6.71 | 100 |  |  |
| Pk YNWS | 1 | 2.08 ± 0.29 | 8.53 ± 0.06 | 10.61 ± 0.24 | 4.16 ± 0.66 | st |
|  | 2 | 2.07 ± 0.11 | 8.48 ± 0.17 | 10.55 ± 0.21 | 4.11 ± 0.24 | st |
|  | 3 | 2.23 ± 0.27 | 7.77 ± 0.14 | 9.99 ± 0.37 | 3.52 ± 0.38 | st^†^ |
|  | 4 | 1.73 ± 0.33 | 7.75 ± 0.30 | 9.48 ± 0.42 | 4.59 ± 0.84 | st |
|  | 5 | 3.33 ± 0.25 | 5.74 ± 0.37 | 9.07 ± 0.58 | 1.73 ± 0.10 | sm |
|  | 6 | 1.59 ± 0.32 | 6.82 ± 0.59 | 8.41 ± 0.87 | 4.37 ± 0.58 | st |
|  | 7 | 1.49 ± 0.17 | 5.66 ± 0.37 | 7.15 ± 0.34 | 3.83 ± 0.60 | st |
|  | 8 | 1.98 ± 0.13 | 4.00 ± 0.16 | 5.98 ± 0.29 | 2.02 ± 0.07 | sm^†^ |
|  | 9 | 2.53 ± 0.10 | 2.99 ± 0.36 | 5.52 ± 0.44 | 1.18 ± 0.11 | m |
|  | 10 | 1.50 ± 0.25 | 3.17 ± 0.44 | 4.67 ± 0.23 | 2.19 ± 0.64 | sm |
|  | 11 | 1.39 ± 0.12 | 3.27 ± 0.04 | 4.66 ± 0.08 | 2.36 ± 0.21 | sm |
|  | 12 | 1.64 ± 0.11 | 2.09 ± 0.24 | 3.73 ± 0.33 | 1.28 ± 0.09 | m |
|  | 13 | 1.46 ± 0.15 | 1.99 ± 0.09 | 3.45 ± 0.14 | 1.38 ± 0.18 | m |
|  | 14 | 1.46 ± 0.05 | 1.93 ± 0.20 | 3.39 ± 0.25 | 1.31 ± 0.09 | m |
|  | 15 | 1.42 ± 0.16 | 1.92 ± 0.14 | 3.34 ± 0.27 | 1.36 ± 0.12 | m |
|  | total | 27.89 ± 2.82 | 72.11 ± 3.67 | 100 |  |  |
| Po HNXH | 1 | 5.74 ± 0.46 | 8.22 ± 0.12 | 13.96 ± 0.58 | 1.44 ± 0.09 | m |
|  | 2 | 4.86 ± 0.16 | 7.67 ± 0.28 | 12.52 ± 0.31 | 1.58 ± 0.08 | m^†^ |
|  | 3 | 3.51 ± 0.42 | 8.79 ± 0.09 | 12.29 ± 0.51 | 2.53 ± 0.27 | sm |
|  | 4 | 3.32 ± 0.04 | 8.94 ± 0.13 | 12.26 ± 0.17 | 2.69 ± 0.02 | sm |
|  | 5 | 2.93 ± 0.12 | 8.68 ± 0.09 | 11.61 ± 0.19 | 2.97 ± 0.11 | sm^†^ |
|  | 6 | 2.95 ± 0.35 | 8.62 ± 0.28 | 11.57 ± 0.07 | 2.96 ± 0.47 | sm |
|  | 7 | 2.93 ± 0.28 | 3.88 ± 0.24 | 6.81 ± 0.51 | 1.33 ± 0.05 | m |
|  | 8 | 2.99 ± 0.09 | 3.69 ± 0.19 | 6.68 ± 0.25 | 1.23 ± 0.06 | m |
|  | 9 | 2.34 ± 0.10 | 4.01 ± 0.06 | 6.35 ± 0.11 | 1.72 ± 0.08 | sm |
|  | 10 | 1.65 ± 0.11 | 4.30 ± 0.25 | 5.95± 0.26 | 2.61 ± 0.25 | sm |
|  | Total | 33.21 ± 2.14 | 66.79 ± 1.72 | 100 |  |  |
| Po AHDBS | 1 | 3.44 ± 0.33 | 9.75 ± 0.24 | 13.20 ± 0.17 | 2.86 ± 0.33 | sm |
|  | 2 | 2.86 ± 0.58 | 9.96 ± 0.71 | 12.82 ± 0.68 | 3.58 ± 0.78 | st^†^ |
|  | 3 | 3.36 ± 0.29 | 9.03 ± 0.21 | 12.39 ± 0.50 | 2.70 ± 0.18 | sm |
|  | 4 | 2.04 ± 0.18 | 10.03 ± 0.23 | 12.08 ± 0.18 | 4.94 ± 0.51 | st |
|  | 5 | 4.24 ± 0.21 | 7.77 ± 0.50 | 12.01 ± 0.71 | 1.83 ± 0.03 | sm^†^ |
|  | 6 | 2.89 ± 0.08 | 4.38 ± 0.68 | 7.27 ± 0.66 | 1.52 ± 0.25 | m |
|  | 7 | 2.86 ± 0.08 | 4.04 ± 0.15 | 6.90 ± 0.23 | 1.41 ± 0.02 | m |
|  | 8 | 1.48 ± 0.05 | 5.12 ± 0.28 | 6.60 ± 0.25 | 3.48 ± 0.29 | st |
|  | 9 | 2.65 ± 0.15 | 3.39 ± 0.25 | 6.04 ± 0.40 | 1.28 ± 0.02 | m |
|  | 10 | 2.06 ± 0.23 | 3.73 ± 0.21 | 5.79 ± 0.02 | 1.83 ± 0.30 | sm |
|  | 11 | 1.42 ± 0.06 | 3.48 ± 0.18 | 4.90 ± 0.13 | 2.45 ± 0.22 | sm |
|  | Total | 29.31± 2.24 | 70.69 ± 3.65 | 100 |  |  |
| Ps HNFNS | 1 | 3.91 ± 0.23 | 7.25 ± 0.39 | 11.16 ± 0.54 | 1.86 ± 0.11 | sm^†^ |
|  | 2 | 2.66 ± 0.14 | 8.09 ± 0.39 | 10.76 ± 0.25 | 3.05 ± 0.30 | st |
|  | 3 | 2.28 ± 0.30 | 7.91 ± 0.26 | 10.19 ± 0.31 | 3.51 ± 0.52 | st^†^ |
|  | 4 | 2.24 ± 0.35 | 7.61 ± 0.49 | 9.85 ± 0.55 | 3.45 ± 0.58 | st |
|  | 5 | 2.25 ± 0.60 | 6.88 ± 0.24 | 9.14 ± 0.76 | 3.21 ± 0.90 | st |
|  | 6 | 1.85 ± 0.59 | 6.96 ± 0.59 | 8.81 ± 0.14 | 4.07 ± 1.43 | st |
|  | 7 | 1.84 ± 0.14 | 6.56 ± 0.35 | 8.40 ± 0.22 | 3.59 ± 0.46 | st |
|  | 8 | 2.32 ± 0.89 | 4.54 ± 0.68 | 6.86 ± 0.31 | 2.30 ± 1.37 | sm |
|  | 9 | 2.00 ± 0.51 | 4.40 ± 0.99 | 6.39 ± 0.82 | 2.37 ± 1.14 | sm |
|  | 10 | 2.23 ± 0.22 | 4.09 ± 0.59 | 6.32 ± 0.47 | 1.86 ± 0.40 | sm |
|  | 11 | 2.18 ± 0.05 | 4.04 ± 0.34 | 6.22 ± 0.34 | 1.85 ± 0.17 | sm |
|  | 12 | 1.87 ± 0.42 | 4.03 ± 0.33 | 5.91 ±0.39 | 2.24 ± 0.61 | sm |
|  | Total | 27.64 ± 4.43 | 72.36 ± 5.66 | 100 |  |  |

^※^Pc = *P. cyrtonema,* Pk = *P. kingianum,* Po = *P. odoratum,* Ps = *P. sibiricum.*

†satellite chromosome (satellite length was included in chromosome length but the length of secondary constriction was excluded).
