# Supplementary material for: ﻿Molecular cytogenetic characterization of 9 populations of four species in the genus Polygonatum (Asparagaceae)
Source: Comp Cytogenet. 2024 May 16;18:73–95. doi: 10.3897/compcytogen.18.122399 (PMC11116888; doi:10.3897/compcytogen.18.122399)
Supplement: Supplementary material 3 — Metaphase chromosomes of 9 populations of four Polygonatum species, P.cyrtonema (Pc), P.kingianum (Pk), P.odoratum (Po) and P.sibiricum (Ps) [file comparative_cytogenetics-18-073_article-122399__-s003.docx]

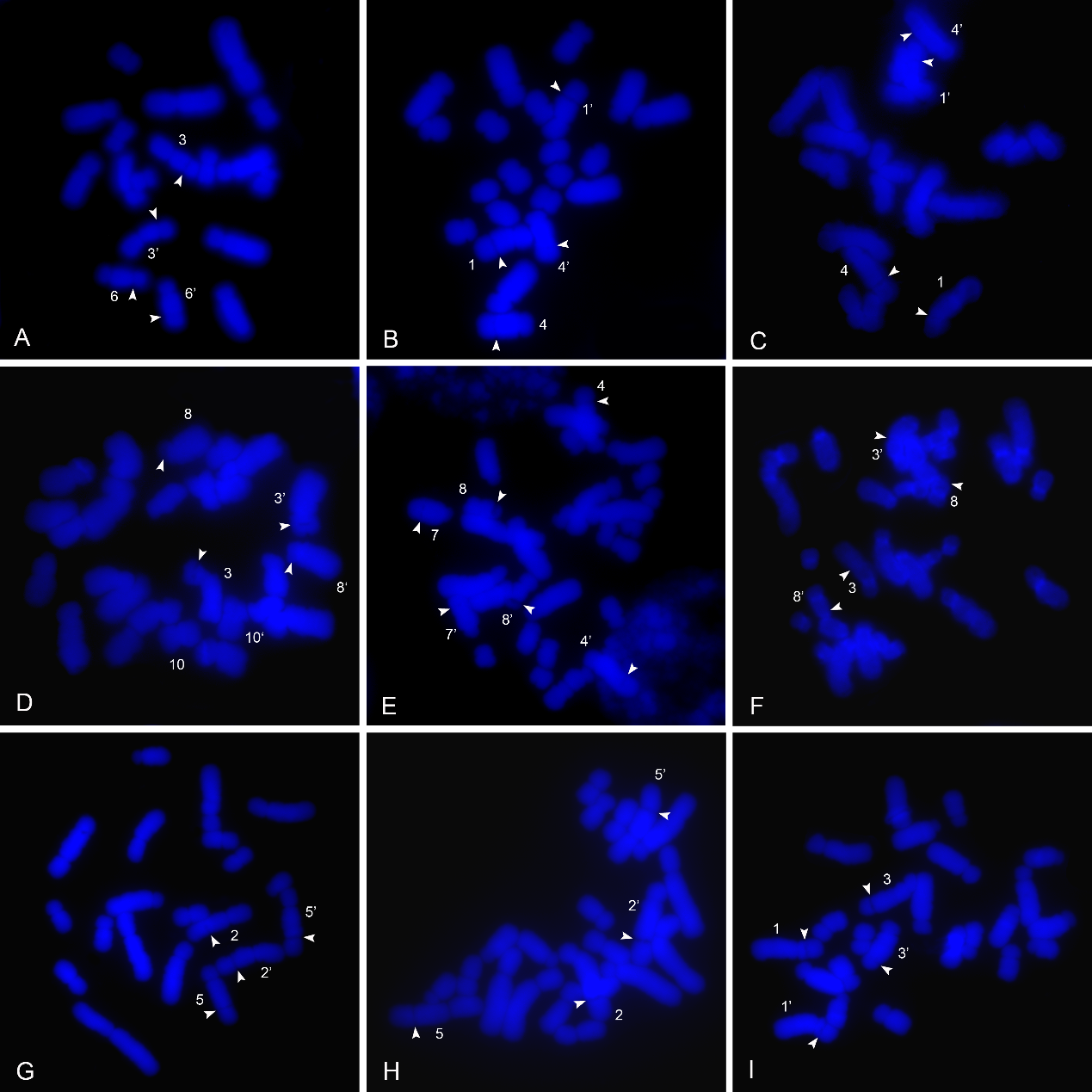


**Figure S1.** Metaphase chromosomes of 9 populations of four *Polygonatum* species, *P. cyrtonema* (Pc), *P. kingianum* (Pk), *P. odoratum* (Po) and *P. sibiricum* (Ps). The chromosomes were counterstained with DAPI (blue). Arrowheads indicate the secondary constrictions (SCs). The numbers indicate the serial number of the chromosomes bearing SCs. **A** Pc AHDBS, **B** Pc HNHH, **C** Pc HBHS, **D** Pc SCSN, **E** Pk YNKM, **F** Pk YNWS, **G** Po HNXH, **H** Po AHDBS, **I** Ps HNFNS.
