# Supplementary material for: ﻿Molecular cytogenetic characterization of 9 populations of four species in the genus Polygonatum (Asparagaceae)
Source: Comp Cytogenet. 2024 May 16;18:73–95. doi: 10.3897/compcytogen.18.122399 (PMC11116888; doi:10.3897/compcytogen.18.122399)
Supplement: Supplementary material 4 — Diagrams of the distribution of basic chromosome numbers within four Polygonatum species, P.cyrtonema, P.kingianum, P.odoratum and P.sibiricum [file comparative_cytogenetics-18-073_article-122399__-s004.docx]

**
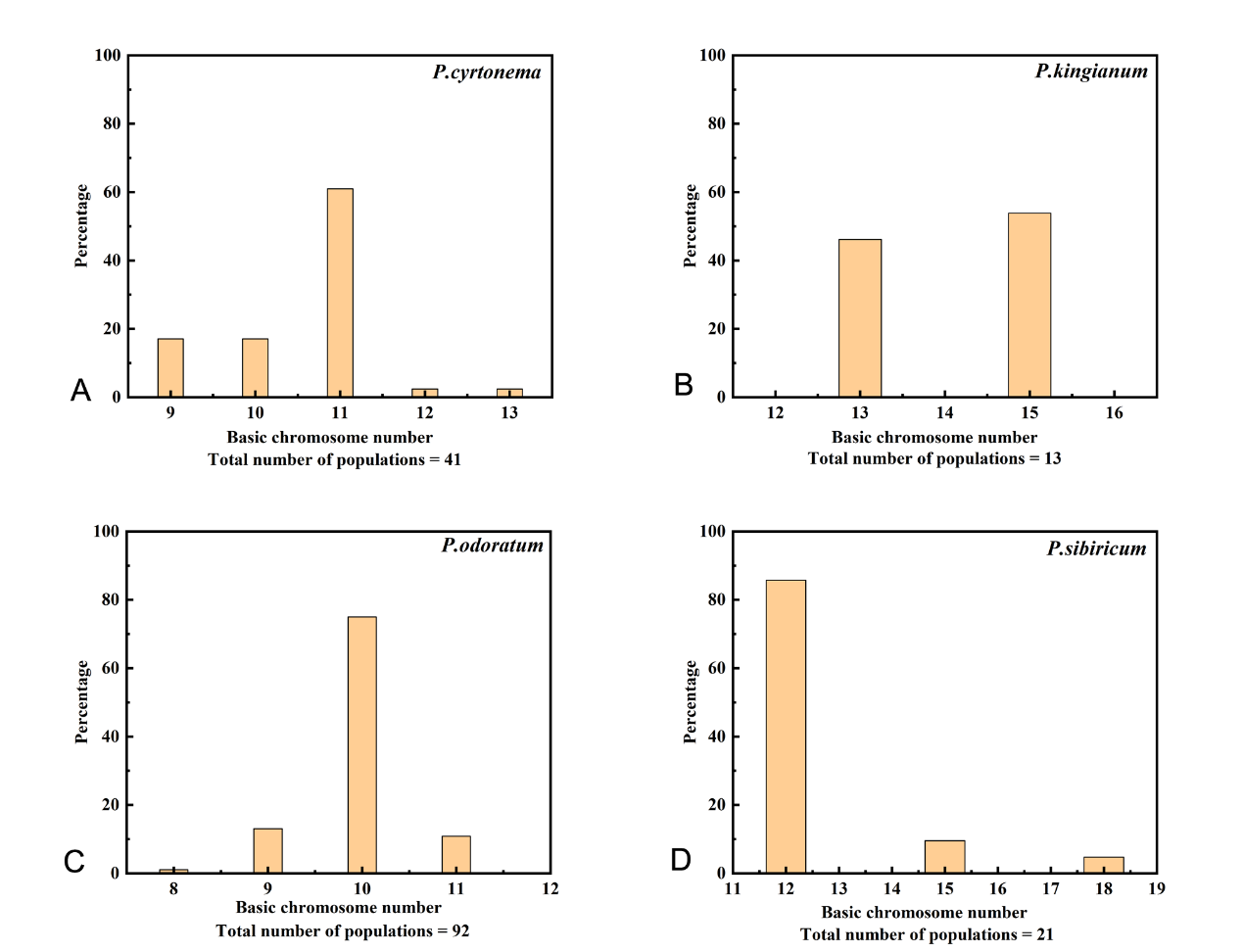
**

**Figure S2.** Diagrams of the distribution of basic chromosome numbers within four *Polygonatum* species, *P. cyrtonema*, *P. kingianum*, *P. odoratum* and *P. sibiricum*. The statistics were based on the chromosome counts of the diploid populations reported in the literature and used in this study.
